# Arc-discharge-assembled CNT/MoO_3_ nanohybrids for ultra-sensitive and selective sub-ppm ethanol detection at room temperature

**DOI:** 10.1039/d6ra00372a

**Published:** 2026-03-19

**Authors:** Hieu Minh Nguyen, Phuoc Van Cao, Anh Viet Cao, Hung Manh Nguyen, Chinh Duc Nguyen, Trieu Hai Vu, Sutripto Majumder, Chuc Gia Hoang, Minh Van Do, Trung Quang Do, Tu Nguyen, Du Van Nguyen, Trung Manh Tran, Huy Thanh Pham, Jong-Ryul Jeong, Chunjoong Kim, Dojin Kim

**Affiliations:** a Faculty of Materials Science and Engineering, PHENIKAA School of Engineering, PHENIKAA University Nguyen Trac, Duong Noi Hanoi 12116 Vietnam hieu.nguyenminh@phenikaa-uni.edu.vn; b Department of Materials Science and Engineering, Chungnam National University Daejeon 34134 Republic of Korea; c Department of Mechanical Engineering, Chungnam National University Daejeon 34134 Republic of Korea; d Faculty of Fundamental Sciences, PHENIKAA School of Engineering, PHENIKAA University Nguyen Trac, Duong Noi Hanoi 12116 Vietnam; e Department of Materials Science and Engineering, Le Quy Don Technical University Hanoi 100000 Vietnam; f Saveetha School of Engineering, Saveetha Institute of Medical and Technical Sciences (SIMATS) Chennai-602105 Tamil Nadu India

## Abstract

In this study, one-dimensional carbon nanotube (CNT) and zero-dimensional MoO_3_ nanohybrids were synthesized using a simple arc-discharge method for ethanol gas sensor applications. MoO_3_ nanoparticles were uniformly distributed on the surface of mesoporous CNTs, which increased the specific surface area and the availability of active sites for charge carriers within the nanohybrid. MoO_3_ functions as the receptor, while the CNTs serve as the transducer, leading to the modification in the depletion region at the hybrid surface, followed by enhancement of the sensing performance. The CNT/MoO_3_ sensor exhibited the highest response of 76.5% to 1 ppm ethanol even at room temperature operation (30 °C), significantly outperforming CNT (12.5%) and MoO_3_ (2.5%). Additionally, the CNT/MoO_3_ sensor revealed rapid response and recovery time, excellent selectivity, and minimal humidity dependence. SEM, TEM, XRD, XPS, and BET analyses confirmed that the improved gas sensitivity of the CNT/MoO_3_ nanohybrid is attributed to the increased active sites for charge carriers, abundant surface vacancies, and modification in the depletion region.

## Abstract 2

(1) This is the first study of CNT/MoO_3_ for ethanol sensing detection at room temperature with rapid response and recovery times. (2) The commonly accepted chemisorbed oxygen theory no longer plays a dominant role in the sensing mechanism.

## Graphical abstract

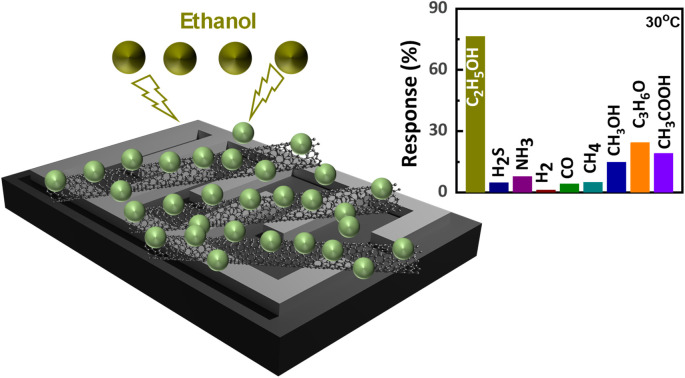

## 1. Introduction

Volatile organic compounds (VOCs) have long been recognized as significant threats to environmental safety and human health.^1^ Among these, ethanol, the most common VOC, poses a serious health risk at room temperature (RT) when prolonged exposure to ambient air exists.^2–4^ Therefore, there is a strong demand for the development of highly sensitive, small-sized, fast-response materials with enhanced selectivity and long-term stability at RT to protect human health.^5^ Metal oxide semiconductors (MOSs) are widely used as the primary sensing materials due to their excellent stability, low cost, and good response characteristics.^6,7^ Therefore, studies on MOS-based gas sensors, such as ZnO,^8–10^ In_2_O_3_,^11,12^ Fe_2_O_3_,^13^ and MoO_3_^14,15^ have been extensively reported. Among these, MoO_3_, a novel n-type semiconductor, offers advantages such as low production cost and high electron transfer efficiency, making it a promising candidate for use in gas sensors, batteries, and other related applications.^16,17^ For instance, Wang *et al.* synthesized MoO_3_/SnO_2_ core–shell heterostructures that exhibited a high response value toward 100 ppm ethanol at 200 °C (ref. 18) and MoO_3_ prepared by Li *et al.* demonstrated a response of 3.86 toward 20 ppm ethanol exposure at 240 °C, with excellent ethanol selectivity.^19^ However, the high operating temperature hinders the applicability of ethanol detection because of the significant risk of explosion.^20,21^ Therefore, reducing the operating temperature of MoO_3_-based sensors while maintaining good response characteristics, fast response/recovery kinetics, and high selectivity is critical.

In the meantime, carbon nanotubes (CNTs) have been widely explored in the low-temperature sensor application since their discovery by Iijima *et al.* in 1991 (ref. 22) due to their high surface area, large active sites for gas contact, and low operating temperatures.^23^ Hybridization of CNTs with MOSs could introduce synergistic effects,^24,25^ further enhancing the gas sensitivity of the composite material.^7,26,27^ CNTs, which are known to exhibit p-type semiconductor properties,^28^ can form p–n heterojunctions with n-type semiconductors like MoO_3_. Such heterojunctions oftentimes lead to improved gas sensitivity and selectivity while lowering the operating temperature of the sensor.

However, only a few studies about p–n heterojunctions involving CNT/MoO_3_ for ethanol detection at room temperature have been reported. In this study, we successfully fabricated CNT/MoO_3_ hybrid structures with various CNT contents by the arc-discharge method. The hybrid structures were characterized using SEM, TEM, XRD, Raman, XPS, and BET analyses to investigate their morphology, electronic state, specific surface area, surface features, pore volume, and gas adsorption properties. The CNT/MoO_3_ nanohybrid exhibited a higher specific surface area, thereby providing more surface-active sites for ethanol adsorption. The charge carrier transfer mechanism is modified by the formation of a depletion layer in the nanohybrid, leading to high sensing capability. Furthermore, we unveiled the sensing mechanism and the origin of the highest sensing capability in our nanohybrid materials.

## 2. Experimental section

### 2.1. Fabrication of CNT/MoO_3_ hybrid sensors

**Fig. 1:**
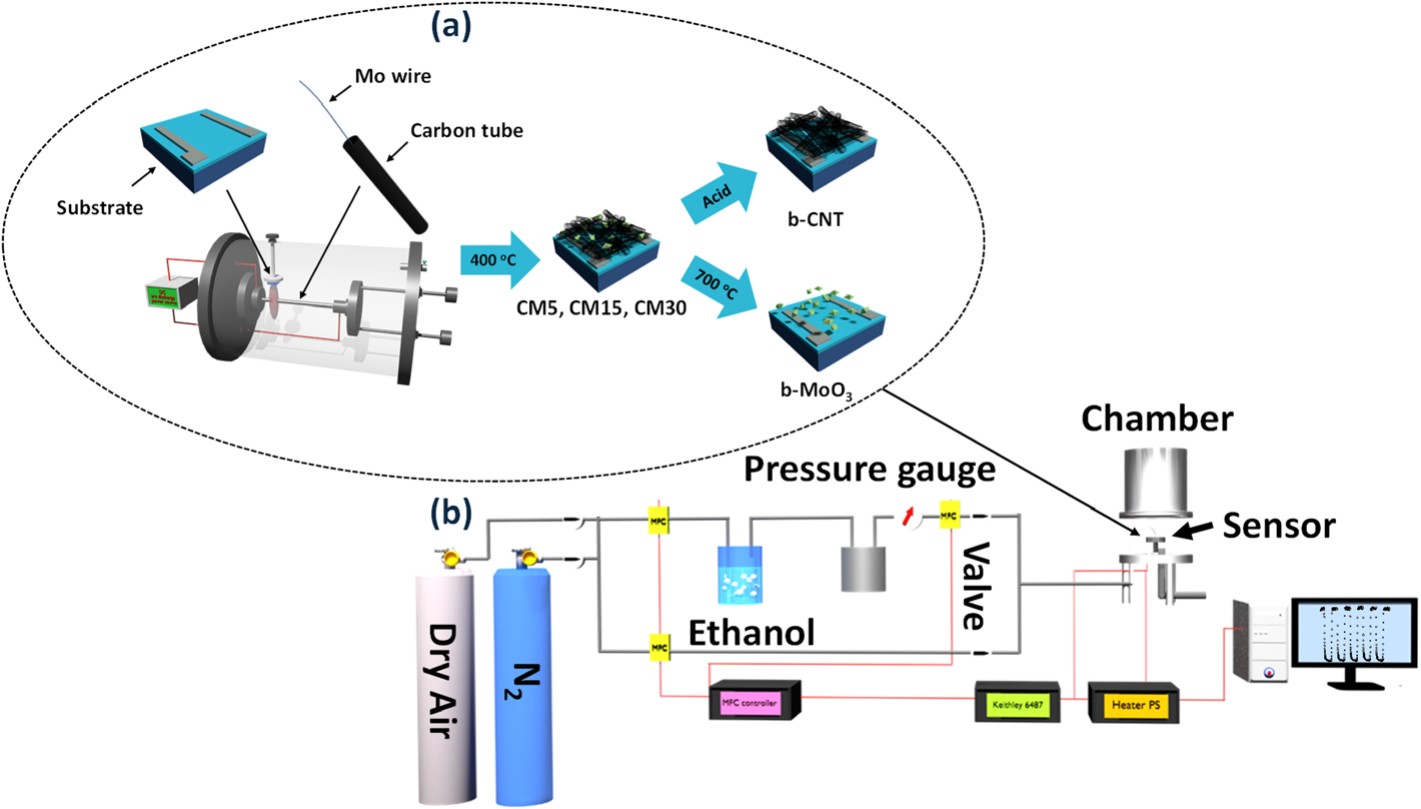
(a) The fabrication flowchart of the CNT/MoO_3_ composite sensor on the Si/SiO_2_ substrate. (b) Experimental setup for gas sensing measurement.

CNT/MoO_3_ hybrid sensors were fabricated on SiO_2_/Si substrates *via* a co-arc discharge method. The substrates (dimensions: 5 mm × 0.65 mm, oxide thickness: 300 nm) underwent ultrasonic cleaning in acetone, methanol, and deionized water for 15 minutes each, followed by drying with nitrogen gas. Titanium and platinum layers with 30 nm- and 120 nm-thickness, respectively, were sequentially deposited to the substrate *via* DC magnetron sputtering, employing a shadow mask to form the parallel-electrode configurations. These electrode-patterned substrates were then placed on the inner wall of the arc-discharge chamber, following the procedure detailed in previous studies.^7,8,26^ The active sensing area was defined using Scotch tape as a mask. The arc discharge was conducted at a current density of 45 A cm^−2^ in a hydrogen (H_2_) atmosphere with a partial pressure of 6.0 × 10^3^ Pa. Five molybdenum wires served as the catalyst source were inserted into a pure hollow graphite tube (inner diameter: 3 mm; outer diameter: 6.4 mm; length: 160 mm). The thickness of the CNT/MoO_3_ nanohybrid was adjusted by varying the co-arc discharge duration to 5, 15, or 30 minutes. To enhance adhesion and ensure reliable electrical contact between the CNT/MoO_3_ nanohybrid and the Si/SiO_2_ substrate, the as-deposited films were immersed in methanol. The resulting films went through a 400 °C heat treatment for 2 hours in dry air to eliminate the remaining amorphous carbon and fully oxidize the molybdenum to MoO_3_.^27^ The samples with 5, 15, and 30 min were designated as CM5, CM15, and CM30, respectively. A schematic flowchart illustrating the CNT/MoO_3_ hybrid sensor synthesis is presented in Fig. 1a. In addition, chemical treatment with a mixture of nitric and sulfuric acid (1 : 3 ratio) was carried out for the CM15 sample to isolate bare CNTs (b-CNT). CM15 samples were also annealed at 700 °C for 3 hours to yield bare MoO_3_ (b-MoO_3_) by burning off CNTs.

### 2.2. Morphological and structural characterization

The morphological characteristics of the CNT/MoO_3_ network structures were analyzed using field-emission scanning electron microscopy (FE-SEM; JSM 700F, JEOL, Japan) and high-resolution transmission electron microscopy (HR-TEM; Tecnai G2 F30 S-TWIN, FEI, Netherlands). The X-ray diffraction was conducted using X'pert PRO-MPD (PANalytical, Netherlands) with Cu Kα radiation. To explore the chemical bonding states, we employed the X-ray photoelectron spectroscopy (XPS; Thermo Scientific MultiLab 2000, Pittsburgh, PA, USA) with monochromatic Al Kα radiation and Raman spectroscopy (UniRAM spectrometer equipped with a 532 nm excitation laser and a cooled CCD detector). Additionally, nitrogen adsorption/desorption analysis was conducted to evaluate the surface area and pore characteristics of the hybrid structure using the Autosorb-1-C analyzer (Quantachrome).

### 2.3. Sensing measurement

Fig. 1b shows the schematic of the gas detection system using a pico-ammeter/voltage (Keithley 6487). The sensor substrates were placed on the top of the chamber in which the mass flow controllers (MFCs) control the sensor operating temperature and gas transfer rate. By applying a 1 V bias between the sensor electrodes, the corresponding resistance and conductance were recorded. Saturated ethanol vapors were produced using a bubbler system, in which dry air was passed through pure ethanol to generate a controlled vapor stream. The Antoine equation was used to determine the vapor pressure, which is given by: 
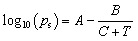
, where *p*_s_ is the saturated vapor pressure, *T* is the temperature of the gas-liquid system^29^ and *A*, *B*, and *C* are component-specific constants. The relative substance concentration in the gas phase (*x*_s_) was determined by *x*_s_ = *Ps*/*P*, where *P* is the total pressure of the entire system. The ethanol gas or and humidity concentration, *C*_g_ and *C*_H_, respectively, was calculated *via* the equation: 
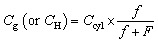
, where *f* and *F* are the flow rates of analyte gas (ethanol) and carrier gas, respectively, while *C*_cyl_ is the analyte gas concentration in the cylinder (or *C*_cyl_ = *x*_s_ for humidity exposure).^30^ The sensor response, *S* (%), is calculated using the equation: (*R*_g_ − *R*_o_)/*R*_o_ × 100, where *R*_o_ and *R*_g_ represent the baseline resistance in dry air before ethanol exposure and the resistance measured during ethanol exposure, respectively. For the gas selectivity test, the sensor was exposed toward 3 ppm of H_2_S, NH_3_, H_2_, CO, CH_4_, methanol, acetone, and acetic acid gases. To operate the system under the humidity effect, water molecules were similarly pumped through the bubbler with the flow rate ratio controlled by the MFCs. The relative humidity index was determined by the Testo 635 Temperature and Moisture meters.

## 3. Results and discussion

### 3.1. Morphology and structure

**Fig. 2:**
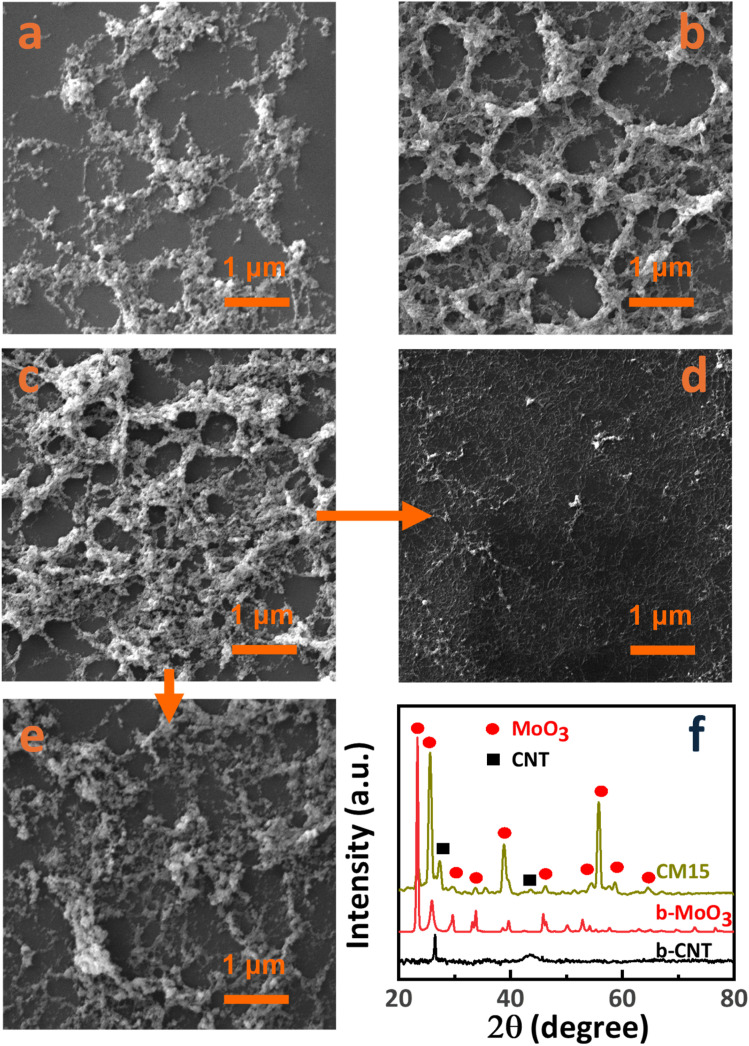
SEM illustration of CNT/MoO_3_ hybrids (a) CM5, (b) CM15, (c) CM30, (d) b-CNT, and (e) b-MoO_3_. (f) XRD patterns of b-CNT, b-MoO_3_, and CM15.

**Fig. 3:**
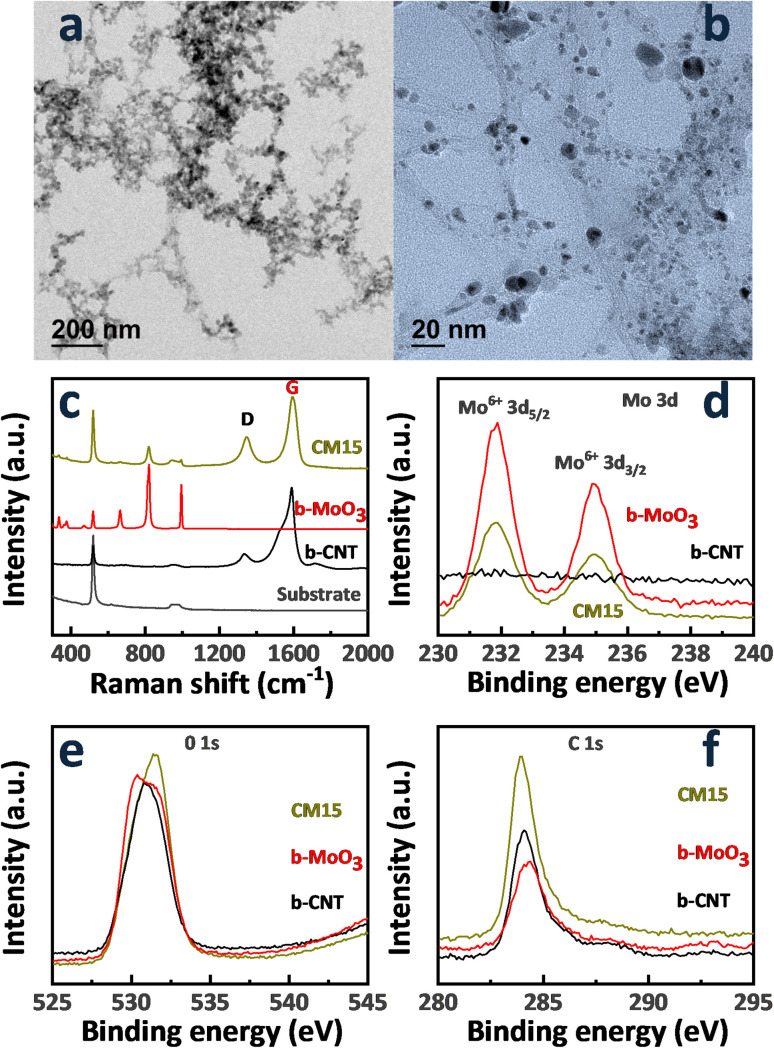
(a) Low and (b) high-magnification TEM images of the CNT/MoO_3_ hybrids (CM15). (c) Raman spectra of b-CNT, b-MoO_3_, and CM15. High-resolution XPS spectra of (d) Mo 3d, (e) O 1s, and (f) C 1s states of b-CNT, b-MoO_3_, and CM15.

The co-arc discharge process with a molybdenum-embedded graphite tube facilitates the simultaneous evaporation of molybdenum and graphite, leading to the formation of Mo nanoparticles and the growth of CNTs, which is primarily driven by the catalytic activity of molybdenum. Molybdenum in the graphite tube resulted in the formation of Mo nanoparticles, with CNT bundles interweaving through these particles. Subsequent oxidation of the as-synthesized Mo-embedded CNTs at 400 °C produced the CNT/MoO_3_ nanohybrid structure. CNT/MoO_3_ nanohybrids with various deposition times (5, 15, and 30 min) were denoted by CM5, CM15, and CM30, respectively, and their SEM images are presented in Fig. 2a, b, and c. The concentration of the CNT/MoO_3_ nanohybrid on the substrate increased progressively with deposition time. SEM images confirmed the porous structure of the CNT/MoO_3_ nanohybrid network, characterized by CNT bundles interwoven with MoO_3_ nanoparticles. A continuous layer covering the entire sensor surface was not achieved even in the CM30 sample. The size of the MoO_3_ particles ranges from several nanometers to tens of nanometers regardless of the deposition time. The SEM images of bare CNT (b-CNT) and bare MoO_3_ (b-MoO_3_) are also presented in Fig. 2d and e, respectively. In Fig. 2d, MoO_3_ particles were completely leached out by acid treatment while preserving only CNTs on the substrate. Meanwhile, CNTs were completely combusted at 700 °C, leaving only MoO_3_. High-resolution TEM images (Fig. 3a and b) further visualize the nanohybrid morphology, clearly confirming entangled MoO_3_ nanoparticles interconnected with the CNT bundle network. TEM analysis also revealed the presence of single-walled CNTs due to the catalytic role of Mo.

The XRD patterns of the CNT/MoO_3_ nanohybrids, along with the bare CNT (b-CNT) and bare MoO_3_ (b-MoO_3_) structures, are presented in Fig. 2f. The characteristic peaks observed at 26.5° and 43° are attributed to the (002) and (100) lattice planes of CNTs, respectively (JCPDS card no. 75-1621). Meanwhile, the peaks at 25.53°, 33.76°, 38.89°, and 55.84° correspond to the (040), (111), (060), and (042) lattice planes of the orthorhombic MoO_3_ phase, respectively (JCPDS card no. 05-0508). Other crystalline phases were not detected. Raman spectra of b-CNT, b-MoO_3_, and CNT/MoO_3_ composite (CM15) between 250 and 2000 cm^−1^ are presented in Fig. 3c. The quality of the CNTs was analyzed by Raman spectroscopy. The sharp peaks indicate that the vibrational modes predominantly result from a well-ordered, high-degree crystalline structure. The Raman corresponding spectrum of CNTs generally consists of two major peaks at ∼1340 cm^−1^ (D band) and ∼1595 cm^−1^ (G band).^31^ The D-band originated by sp^3^ electronic states is well-known for defective, glassy carbon, and disordered graphite, while the G-band is related to the two-dimensional hexagonal lattice sp^2^ vibration in the graphite. The typical high G-band/D-band intensity ratio is a signature of the high crystallinity single-wall CNTs.^32^ The G-band to D-band peak intensity ratios (*I*_G_/*I*_D_) of the CNTs are ∼2 in CM15, demonstrating the good quality of the as-synthesized CNTs. These results demonstrate the structural integrity of sp^2^-hybridized carbon atoms of the single-wall CNTs. The D- and G-band peaks of the b-MoO_3_ disappeared due to burning CNTs out completely at 700 °C. Six typical peaks at 283, 334, 375, 665, 819, and 994 cm^−1^ were observed, which is indicative of the crystal structure of α-MoO_3_. The Raman band observed at 283 cm^−1^ (B_2g_, B_3g_) corresponds to the wagging vibrational modes of the terminal oxygen atoms (Mo=O vibration). The band at 334 cm^−1^ (B_1g_, A_9_) is attributed to the Mo_3_–O bending mode, while the band at 375 cm^−1^ (A_9_) is assigned to the bending mode of the Mo=O bond.^33^ The MoO_6_ octahedral building units contain three nonequivalent oxygen atoms: terminal Mo=O (O_1_), twofold-bridged Mo–O–Mo (O_2_), and threefold-bridged O–Mo_3_ (O_3_). The Raman shifts at 994 cm^−1^ (O_1_), 819 cm^−1^ (O_2_), and 665 cm^−1^ (O_3_) are associated with the stretching vibrations of these respective oxygen sites, characteristic of the α-MoO_3_ crystal structure.^34^ In addition, b-CNT did not show any appearance of peaks related to the α-MoO_3_, confirming the purity of CNTs after acid treatment.

The XPS spectra of b- CNT, b- MoO_3_, and the nanohybrid CM15 are displayed in Fig. 3d, e, and f, respectively. In Fig. 3d, the typical doublet peaks at binding energies of ∼232 eV and ∼235 eV match with 3d_5/2_ and 3d_3/2_ states in Mo^6+^, respectively.^35^ The core-level spectrum of the O 1s state consists of two chemical states: the lower binding energy region (529.8–530.1 eV) corresponding to bulk oxygen anions within the MoO_3_ matrix and the higher binding energy region (530.5–531.0 eV) associated with accumulated oxide impurities. The C 1s spectrum, Fig. 3f, exhibits an asymmetric feature, which can be deconvoluted into two peaks. The dominant peak at ∼284.5 eV stems from carbon–carbon interactions, including sp^2^ hybridized C=C bonds, while the relatively weaker peak at ∼286.6 eV is attributed to the carbon–oxygen interactions, including C–O bonds.^36^

### 3.2. Electrical and gas sensing property

**Fig. 4:**
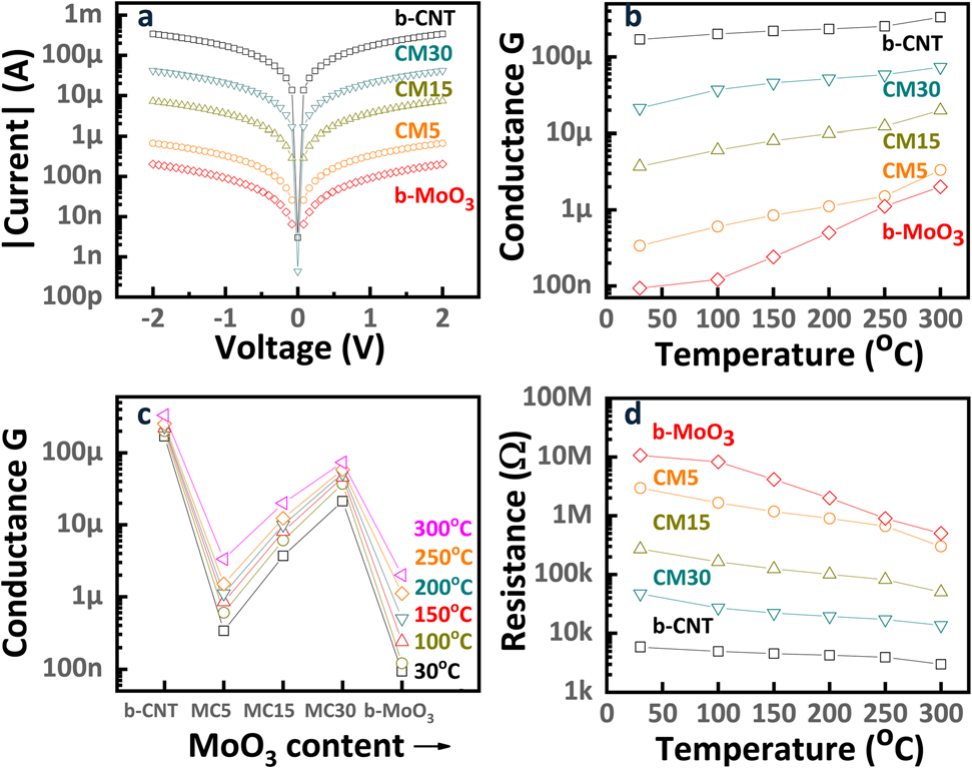
(a) Current–voltage (*I*–*V*) characteristics of b-CNT, b-MoO_3_, CM5, CM15, and CM30 sensors at RT. (b) The conductance of the sensors from 30 °C to 300 °C. (c) The conductance of the sensors is displayed as a function of increasing MoO_3_ content and measuring temperatures. (d) The resistance behavior of the sensors from 30 °C to 300 °C.

The current–voltage (*I*–*V*) characteristics of b-CNT, b-MoO_3_, and CNT/MoO_3_ nanohybrid sensors, Fig. 4a, were evaluated at room temperature (30 °C) under a dry air environment. To ensure accurate measurement, the sensing chamber was pre-heated to 300 °C in dry air to remove any residual water molecules from the sensor surfaces, followed by cooling to room temperature. The *I*–*V* curves, plotted in a logarithmic scale, exhibit consistent ohmic behavior across all sensors. The temperature-dependent conductance (*G*) of the sensors was assessed within the range of 30 °C to 300 °C. Conductance values were calculated using Ohm's relation *I* = *G* × *V*, as depicted in Fig. 4b. The conductance of all sensors increased with temperature, corroborating the semiconducting properties of the materials. Among the samples, b-CNT showed the highest conductance or lowest resistance (Fig. 4d), resulting from the intrinsically high electrical conductivity of CNT as a well-established p-type semiconductor.^37–39^ Meanwhile, MoO_3_ displayed n-type semiconducting behavior with a wide bandgap energy ranging from 3.11 to 3.22 eV.^40,41^ Therefore, in the CNT/MoO_3_ nanohybrid, the conduction mechanism can be explained by a parallel circuit model, where the high-resistance MoO_3_ component and the low-resistance CNTs act as parallel conductive pathways. The overall conductance of the hybrid structure was predominantly influenced by the intrinsically high hole-carrier concentration of CNTs. The incorporation of MoO_3_ into the CNTs network reduced the overall conductance of the composite by several orders of magnitude. The decreasing conductance was likely attributed to the formation of the depletion region at the junction between MoO_3_ particles and CNTs bundles, which effectively acted as an insulating layer and suppressed conductance.^42,43^ The conductance of the nanohybrid increased with greater deposition time, as shown in Fig. 4c, possibly owing to the increased material content. The CM5 sensor, with a thinner CNT/MoO_3_ layer, exhibited higher conductance than the bare MoO_3_ sensor, further underscoring the critical role of CNTs in dictating the electrical behavior of the composite.

**Fig. 5:**
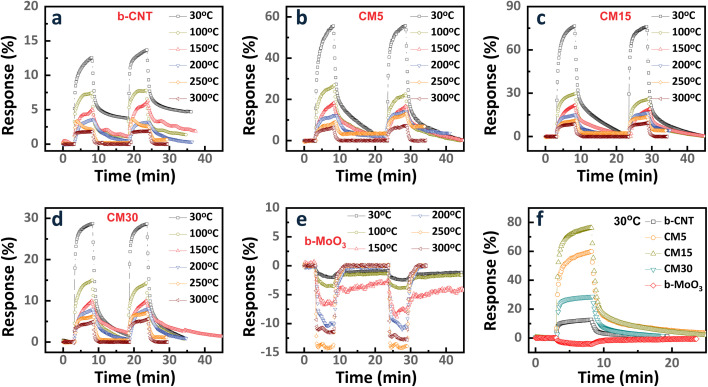
The response and recovery kinetic curves of (a) b-CNT, (b) CM5, (c) CM15, (d) CM30, and (e) b-MoO_3_ sensors to 1 ppm ethanol were measured at varying operating temperatures ranging from 30 °C to 300 °C. (f) The sensor response to 1 ppm ethanol as a function of MoO_3_ content at 30 °C.

**Fig. 6:**
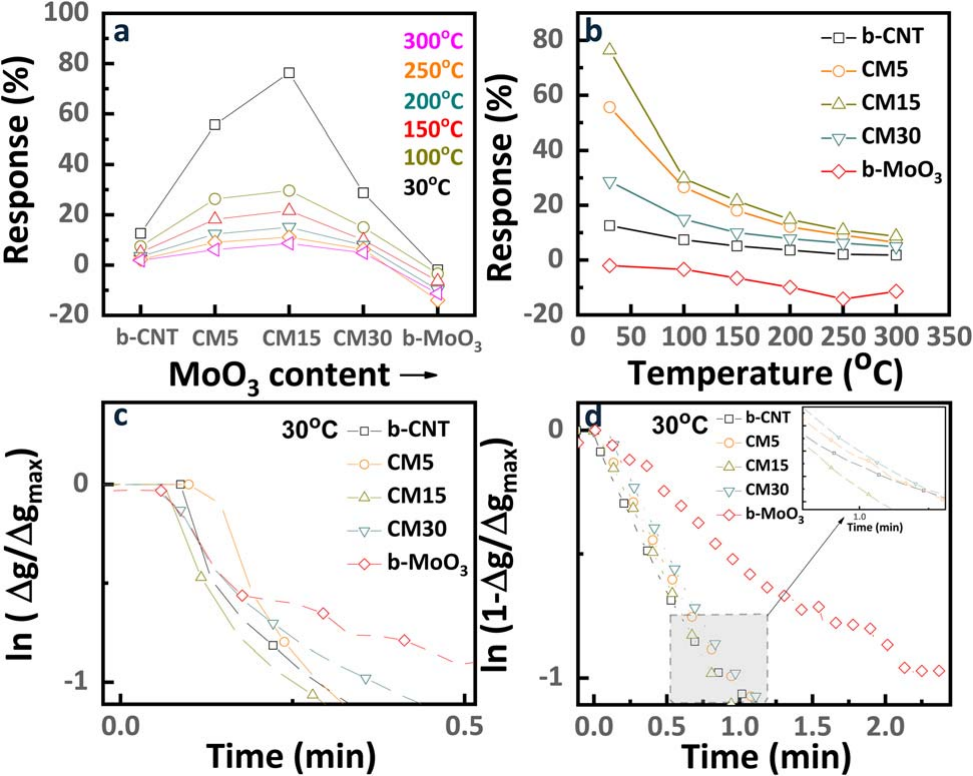
(a) Response/recovery kinetics at 30 °C and (b) summary of response levels at different operating temperatures from 30 °C to 300 °C of b-CNT, CM5, CM15, CM30, and b-MoO_3_ sensors to 1 ppm ethanol. Plots of (c) response time and (d) recovery time following the exponential rise and decay behaviors of the response and recovery kinetics.^44,45^

The ethanol sensing behaviors of the sensors were investigated from 30 °C to 300 °C. The response and recovery kinetics of b-CNT, b-MoO_3_, and CNT/MoO_3_ nanohybrids upon exposure to 1 ppm ethanol in dry air are depicted in Fig. 5. Considering the sensor response S, samples can be categorized by the positive response group (Fig. 5a–d for b-CNT, CM5, CM15, CM30) and the negative response group (Fig. 5e for b-MoO_3_). Since the major charge carriers determine the sensing polarity, the former group shows a *CNT-dominated* conduction mechanism with holes as major charge carriers (p-type). Meanwhile, *MoO*_*3*_*-dominated* conduction with electrons as main charge carriers (n-type) occurs in the latter group. After exposure to ethanol (a reducing gas), the increasing resistance is considered to be a p-type semiconducting behavior. Therefore, the b-CNT, CM5, CM15, and CM30 sensors reveal CNT*-dominated* conduction. Whereas the resistance of the b-MoO_3_ sensor decreases upon exposure to ethanol gas, indicating that the n-type conduction process is predominantly governed by the MoO_3_ component. The sensor responses exhibit rapid kinetics, which reaches sensing saturation within 5 minutes. The behavior of response dependence was replotted in Fig. 5f and 6a to analyze the effect of MoO_3_ content on response systematically. As the b-CNT and CNT-MoO_3_ hybrid sensors operate at a higher temperature, less response can be achieved (Fig. 6b). In contrast, the MoO_3_-dominated sensor, b-MoO_3_, showed the opposite tendency. This difference originates from the difference in sensing mechanisms of CNTs and MoO_3_.

However, the highest sensitivity of the b-MoO_3_ was recorded at 250 °C, which is a general observation for most oxide semiconductors. As shown in Fig. 6a and b, b-CNT and b-MoO_3_ revealed a small response of 12.5% and 4% at RT, respectively. Interestingly, forming the nanohybrids with MoO_3_ and CNTs led to a synergistic effect with the highest response level obtained for the CM15, with a response level of 76.4% at RT, which is much higher than the b-CNT and b-MoO_3_ sensors. The summary of response/recovery curves of the sensors at RT is plotted in Fig. 5f.

The response time and recovery time of the five sensors are displayed in Fig. 6c and d. The CM15 sensor shows the fastest behavior of 14.4 seconds and 66 seconds in both response and recovery time, respectively, compared with other sensors. In addition, Table 1 lists the comparison between this study and other materials along with their corresponding response toward ethanol exposure.

**Table 1:** Gas sensitivity of different sensing materials toward ethanol exposure in literature and this work

Sensor	Concentration (ppm)	Operating temperature (^o^C)	Response (%)	Response time/recovery time (s)	Ref.
MoO_3_/rGO nanoflakes	100	310	3700	4/13	46
Zn/MoO_3_ nanobelts	250	240	4900	48/76	47
MoO_3_/MXene nanocomposites	50	100	453	46/276	48
MoO_3_ nanoplate	100	400	1200	22/15	49
MoO_3_ micro rods	500	332	750	10/10	50
MoO_3_ nanorods/SnO_2_ nanosheets	100	200	4764	65/230	18
MoO_2_/MoO_3_/MXene nanocomposites	200	RT	1870	42/297	51
**CNT/MoO**_**3**_ **nanohybrids**	**1**	**RT**	**76.5**	**14/66**	**This work**

**Fig. 7:**
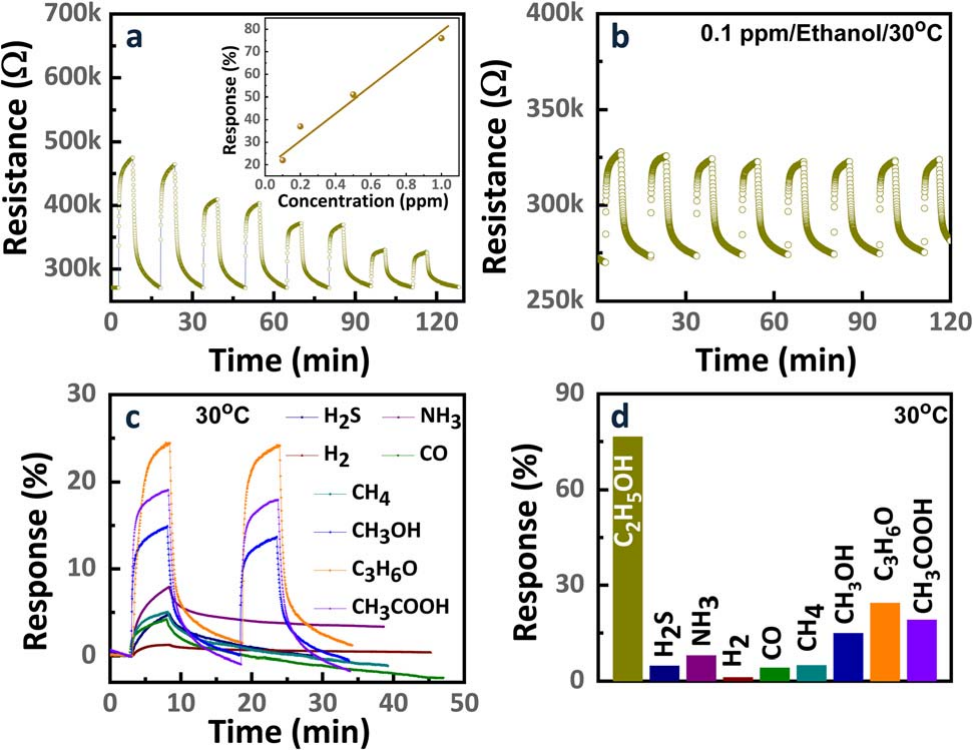
(a) Sensing response/recovery curves of the CM15 sensor against various ethanol concentrations (0.1, 0.2, 0.5, and 1 ppm), and summary of response levels in the inset. (b) Repeatability for 8 cycles of the CM15 sensor for 0.1 ppm ethanol at 30 °C. (c) Response kinetics of the CM15 sensor to 3 ppm of H_2_S, NH_3_, H_2_, CO, CH_4_, methanol, acetone, and acetic acid gases at RT, and (d) comparison of response levels with ethanol sensing.

Given that the CM15 hybrid sensor demonstrated the highest ethanol sensing performance at RT, its sensing behavior was further analyzed across various ethanol gas concentrations (0.1, 0.2, 0.5, and 1 ppm) under the same conditions. As depicted in Fig. 7 and its inset, the sensor exhibited a linear response with respect to ethanol concentration, validating the reliability and consistency of the sensing performance. The linear response can be provided due to enough active surface sites to facilitate the adsorption of ethanol molecules and ensure proportionality in the sensing mechanism.^52^ In addition, Fig. 7b shows the multiple repeatable cycles of the CM15 sensor upon 100 ppb of ethanol. The good repeatability of the CM15 sensor was attained even upon exposure to the low concentration of ethanol, 100 ppb. Finally, the response kinetics of the CM15 sensor to 3 ppm of H_2_S, NH_3_, H_2_, CO, CH_4_, methanol, acetone, and acetic acid gases at RT, and a comparison of its response levels with ethanol sensing are presented in Fig. 7c and d, respectively. The Cn NT/MoO_3_ nanohybrid exhibits good selectivity for ethanol gas detection. The detection performance of ethanol is governed by factors such as dissociation energy, operating temperature, and the properties of the sensing material, including surface defects, morphology, and chemical composition. Accordingly, further optimization of the CNT/MoO_3_ nanohybrid sensor can lead to enhanced performance by fine-tuning these critical parameters.

### 3.3. Gas sensing mechanism

The sensing signal is determined by the chemical adsorption reaction between the ethanol molecules and the pre-adsorbed oxygen ions on the sensor material surface. This interaction modulates the carrier concentration, resulting in changes to the electrical conductivity of the sensors. Initially, negative-charged chemisorbed oxygen ions (O_2_^−^) can be formed through the adsorbed oxygen molecules on the surface of the materials by capturing free electrons from the conduction band of the sensing materials (eqn (1)). Thereby, a depletion layer is generated on the surface of MoO_3_ nanoparticles due to low surface electron density while the oxygen adsorption renders an increase of hole concentration on CNTs surface, which leads to a boost in the conductivity of CNTs. Furthermore, after reducing gas (ethanol) exposure, the removal of chemisorbed oxygen causes the resistance of the p-type dominant CNT-MoO_3_ sensor to increase. As a product of these redox reactions, the trapped electrons released back to the material conduction band and reduced the hole carrier concentration, resulting in a dramatic increase in resistance. Ethanol, a volatile organic compound, can be decomposed by dehydrogenation and dehydration on basic and acidic surface materials,^53–55^ respectively. The methodical decomposition reaction leads to the generation of CO_2_ and H_2_O as presented in eqn (2)–(5).^56–58^ However, the decomposition reactions on the acidic and basic surfaces result in the formation of intermediate products, alkenes, and aldehydes. They originate from bond strength, polar surface, nonpolar surface, anions vacancies, stoichiometric sites, and steric effects of C–H and C–O in ethanol.^53^

1 O_2_ (gas) + e^−^ → O_2_^−^ (adsorbed)

2 C_2_H_5_OH → C_2_H_4_ + H_2_O (acidic surface – hydrogenation)

3 2C_2_H_5_OH → 2CH_3_CHO + H_2_ (basic surface – dehydrogenation)

4 C_2_H_4_ + 3O_2_^−^ → 2CO_2_↑ + 2H_2_O↑ + 3e^−^

5 2CH_3_CHO + 5O_2_^−^ → 4CO_2_↑ + 4H_2_O↑ + 5e^−^

6 H_2_ → 2H_ad_^+^ + 2e^−^ or H_2_ → H_2,ad_^+^ + e^−^

**Fig. 8:**
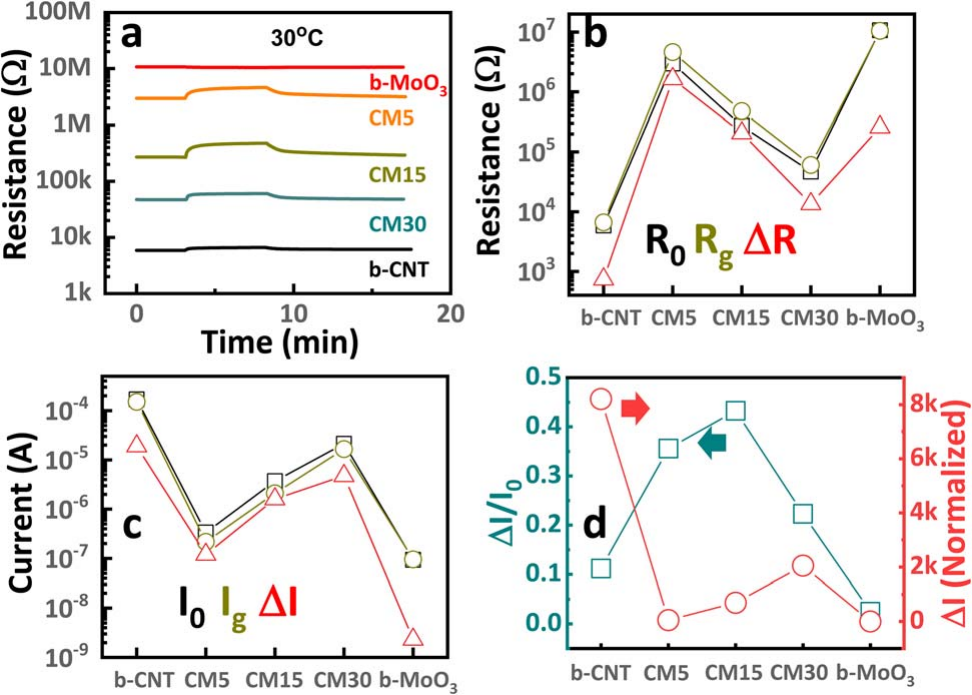
(a) Transient resistance curves of the sensing structures toward 1 ppm ethanol exposure at 30 °C. (b) The corresponding *R*_0_, *R*_g_, and Δ*R*. (c) The corresponding I_0_, *I*_g_, and Δ*I* was converted from (b). (d) The Δ*I*/*I*_0_ values and the Δ*I* normalized to that of b-MoO_3_.

**Fig. 9:**
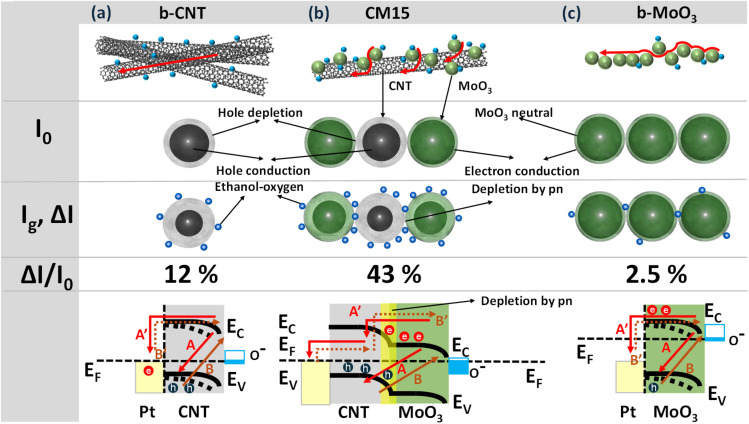
Schematics and energy band diagrams of (a) b-CNT, (b) CM15, and (c) b-MoO_3_ with the modified conductivity and depleted region before (*I*_0_) and after (*I*_g_) 1 ppm ethanol exposure at RT. Blue dots represent the ethanol reaction, while red arrows correspond to the travel distance of main charge carriers.

The released electrons from redox reactions then recombine with CNT holes thus lowering the conductance of the CNT. The charge modulation accompanied by the ethanol adsorption formed the depletion region, as shown in Fig. 9. Upon exposure to 1 ppm ethanol, the resistance of CNTs increases, as indicated by a 12.5% sensing signal at RT. Hence, the resistance further decreases at the elevated temperature (Fig. 5f) owing to the higher rate of ethanol desorption. Meanwhile, b-MoO_3_ shows negligible response to ethanol at room temperature, as illustrated in Fig. 8a. The graph demonstrates that the agglomerated MoO_3_ has neutral cores with minimal surface modification following ethanol exposure, resulting in negligible detectable signal.

As confirmed by our observation, the sensing signal of the hybrid is enhanced with the addition of MoO_3_ on CNTs, especially represented by ∼76.2% in the highest response with CM15 at RT (Fig. 6a). The ethanol sensing signal is defined as the ratio of the sensor resistances before and after ethanol molecule adsorption, denoted as *R*_0_ and *R*_g_, respectively. Fig. 8b presents the raw data of *R*_0_, *R*_g_, and Δ*R* = *R*_g_ − *R*_0_. Alternatively, the data can be expressed in terms of currents *I*_0_ and *I*_g_, and the change in the current Δ*I* = *I*_0_ − *I*_g_, where *I*_0_ and *I*_g_ correspond to the current in the absence and presence of the analyte gas, respectively. This approach is useful because the current is the flow rate of the main charge carriers resulting from the gas–surface interactions. Fig. 8c is derived from resistance (Fig. 8b) using the corresponding current values measured at 1 V. The current variation can be analyzed in two forms: Δ*I*/*I*_0_, which reflects the carrier charge ratio and serves as a measure of sensitivity, and Δ*I*, which indicates the absolute number of carrier charges transferred during the response, normalized to that of b-MoO_3_. The Δ*I*/*I*_0_ value is significantly low for CNTs compared to the hybrid samples. Assuming all charges are consumed for the surface reaction with ethanol, upon ethanol exposure, only 12% of the holes in the CNT participate in the surface reaction. Meanwhile, 35%, 43%, and 22% of the holes in the hybrid materials (CM5, CM15, and CM30, respectively) are involved in the reaction (Fig. 8d). The corresponding number for b-MoO_3_ is only 2.5%. The standby condition of the CM15 with the electron-depleted region of MoO_3_ nanoparticles after contact with CNTs is schemed in Fig. 9b. The improved response of CM15 is attributed to the reduced conductivity of the CNTs, followed by the significant increase in the ethanol adsorption sites at the interference are toward the outer surface. As more MoO_3_ particles contact CNTs, the further expanded junction area results in smaller conductivity in CNTs. In turn, the number of ethanol adsorption sites increases proportionally, ultimately resulting in the complete depletion of CNT holes. Ethanol adsorption is determined by the availability of holes in the CNTs, which restricts further adsorption. Therefore, the higher sensing response of CM15 is attributed to the optimal ratio of conductance before and after ethanol exposure, which corresponds to the highest difference between the initial and post-exposure conductivity states (depicted on the *I*_0_ and *I*_g_, Δ*I* schematics of Fig. 9b). In summary, the incorporation of MoO_3_ into CNT networks enhances the number of active adsorption sites while simultaneously reducing the overall conductivity of the hybrid, achieving the maximum response in CM15. However, with further MoO_3_ additions like CM30, the excessive MoO_3_ content renders adsorption sites over-saturated. The excessively large numbers of MoO_3_ particles are electrically isolated from the CNTs network, thereby disturbing the charge conduction of the hybrids. Moreover, the complete depletion of holes from the CNTs under this condition results in a significant drop in sensing performance. Only ∼20% sensing response could be attained with the CM30 hybrid. Moreover, after ethanol exposure, the released electrons recombined with the holes in CNTs (process A) or moved through the surface route to Pt electrodes (process A′) (Fig. 9a). However, process A hardly occurred due to the restricted number of available holes in CNTs and the low probability of intensive migration through particles. Hence, only 12% of the holes in CNT could be depleted during the gas reaction. In the recovery state, the electrons will be provided either by the electron–hole pair generation process (B) or by transporting electrons from Pt electrodes (process B′). Both processes reveal the high energy barrier and thus lead to slow recovery. Meanwhile, the MoO_3_ nanoparticles are attached to the CNT bundles in CM15 as presented in Fig. 9b, in which released electrons from the reactions as well as from MoO_3,_ can easily recombine with the holes of the CNTs. During the recovery process, the electron transfer rate to the CNT/MoO_3_ interference area is also enhanced through band bending and good heterojunction formation between CNT and MoO_3_. Therefore, both the response and recovery behavior of CM15 are significantly improved, as depicted in Fig. 6d. According to reported values, the work function of CNTs (4.95 eV) exceeds that of MoO_3_ (3.95 eV), facilitating electron transfer from MoO_3_ to CNTs at their interface. This electron transfer further enhances the functional properties of hybrids, particularly for applications involving gas sensing.

To further elucidate the sensing mechanism, the ethanol detection ability of CM15 was investigated upon exposure to 1 ppm ethanol diluted in N_2_ (in the absence of oxygen) at RT. The response curve, illustrated in Fig. S1, showed a response of ∼57%, confirming that ethanol adsorption follows the mechanism described in eqn (6). Additionally, this observation corroborates that ethanol adsorption reveals the oxygen-independent adsorption pathway in CNT/MoO_3_ nanohybrids for ethanol sensing at room temperature. Without the above-mentioned methodical decomposition reaction, enhanced ethanol detection is still clearly observed, which is due to the following reasonable explanation.

Firstly, of particular note is that the CNT-dominated and MoO_3_-dominated sensors show a decrease and an increase in sensing response, respectively, as the temperature increases. At RT, the CNTs adsorb ethanol *via*eqn (4)–(6) while MoO_3_ reveals negligible adsorption. However, at the elevated temperature, the ethanol adsorption and the oxygen ionosorption process of the CNTs are small (as illustrated in Fig. 6a and b) while ethanol dominantly reacted with ionosorbed oxygen on MoO_3_. The simultaneous combustion reaction can occur at the elevated temperature as followed by eqn (4) and (5).^59–61^ Thus, the optimum response of b-MoO_3_ can be achieved at 250 °C, which indicates that the hybrid formation with CNTs in MoO_3_-dominated sensors does not result in any significant synergy effect at a high temperature. However, the combustion reaction contributed to the enhancement of the hybrid sensing behavior in the CNT-dominated sensors with an appropriately small number of MoO_3_ nanoparticles. Since b-CNT does not show any response at the elevated temperature, our observation confirms that electrons released during the combustion reaction at the MoO_3_ nanoparticles are transported to the surface of CNTs, subsequently exhibiting p-type semiconductor behavior.

**Fig. 10:**
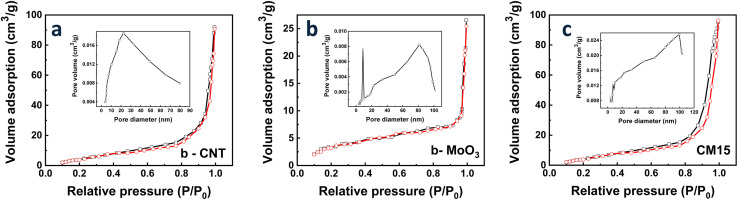
The nitrogen adsorption and desorption isotherms of (a) b-CNT, (b) b-MoO_3_, and (c) CM15 (the insets are the pore size distribution).

Secondly, Fig. 10 presents the nitrogen adsorption–desorption isotherms to analyze the pore size distribution and specific surface area of b-CNT, b-MoO_3_, and CM15. The adsorption–desorption curves exhibit type IV isotherms,^62^ being indicative of mesoporous structures. Based on the Brunauer–Emmett–Teller (BET) equation, the specific surface areas of b-MoO_3_, b-CNT, and CM15 are determined to be 23.1 m^2^ g^−1^, 52.4 m^2^ g^−1^, and 72.12 m^2^ g^−1^, respectively. Additionally, the pore volume of CM15 is measured at 0.073 cm^3^ g^−1^, which is significantly higher than that of b-MoO_3_ (0.013 cm^3^ g^−1^) and b-CNT (0.057 cm^3^ g^−1^). The enhanced specific surface area and pore volume of CM15 suggest an abundance of active sites for oxygen adsorption owing to the hybrid 0D/1D structure of CM15, which is advantageous for gas interaction on the surface. Such improvement directly contributes to enhanced gas detection capabilities.^62^

Thirdly, ethanol exhibits relatively high adsorption energy (*E*_ads_ = −0.96 eV mol^−1^) compared to the adsorption energy of surface-adsorbed oxygen species (*E*_ads_ ∼ −0.35 eV mol^−1^).^63^ This significant difference facilitates preferential interaction between ethanol molecules and the adsorbed oxygen species on the material surface. Such interaction plays a critical role in enhancing the gas detection performance by promoting more effective surface reactions.

Fourthly, the semiconducting characteristics of MoO_3_ on the surface of CNTs serve as an effective catalyst for oxygen dissociation. This catalytic activity boosts the diffusion kinetics of ethanol molecules toward surface vacancies, thereby facilitating rapid surface reactions. As a result, the hybrid material exhibits good sensitivity and fast response to ethanol detection even at room temperature.^64^

And lastly, the exceptional electrical conductivity of CNTs significantly lowers the operating temperature of sensors, enhancing their energy efficiency. Furthermore, the lowest unoccupied molecular orbital (LUMO) energy of ethanol (0.13 eV),^65,66^ accelerates electron transfer between the nanohybrid material and ethanol gas molecules. This facile electron interaction underpins the high sensing selectivity for ethanol compared to other gases.

### 3.4. Sensor performance against humidity effect

**Fig. 11:**
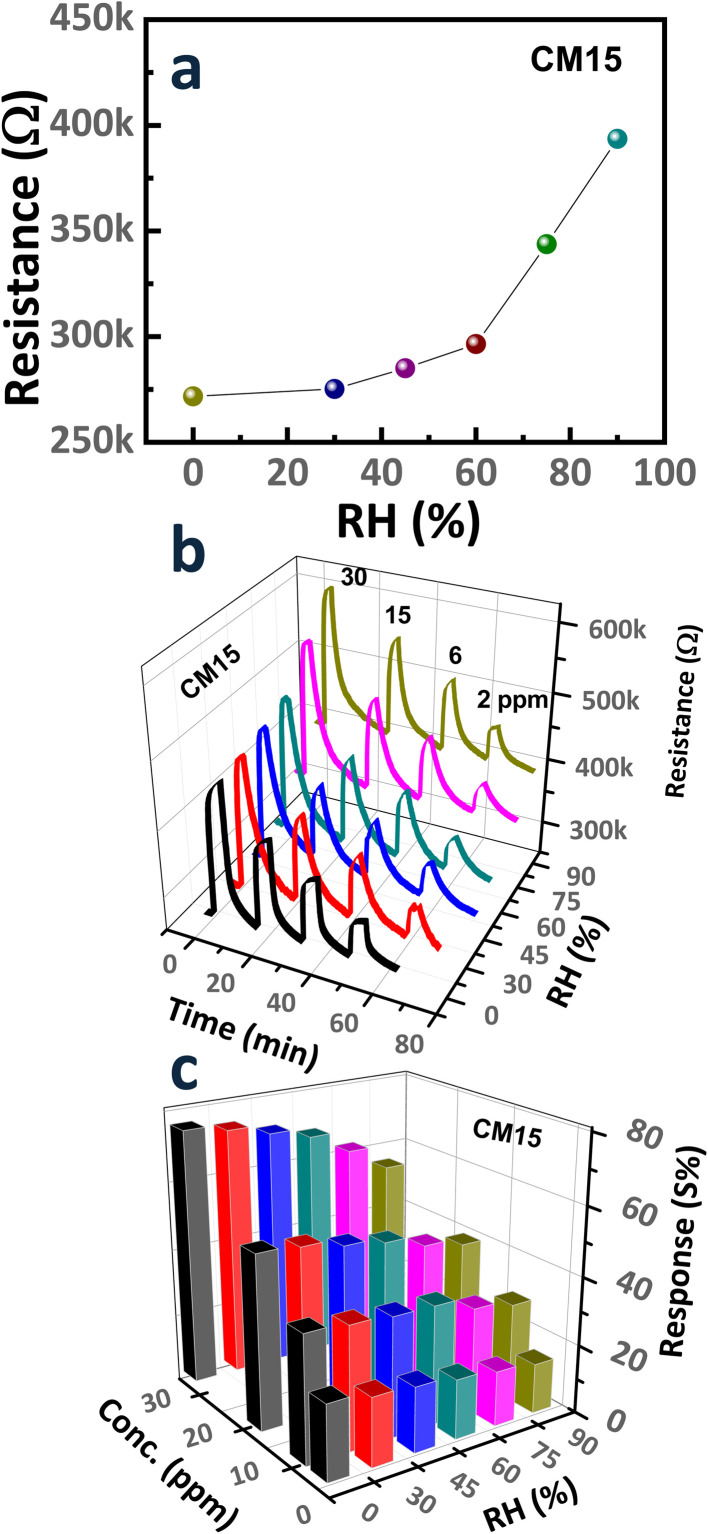
(a) Resistance of the CM15 sensor under varying RH conditions. (b) Transient response curves and (c) corresponding response levels of the CM15 sensor to ethanol concentrations (0.1–1 ppm) at different RH levels (0%, 30%, 45%, 60%, 75%, and 90%) at RT.

For the gas sensor operation at RT, the effect of ambient moisture must be considered. Fig. 11a depicts the influence of relative humidity (RH) on the resistance of sensors, which exhibits a progressive increase in resistance as RH rises from 0% to 90%. Specifically, the baseline resistance of the sensor increased from ∼271.7 kΩ at 0% RH to ∼393.6 kΩ at 90% RH, thereby deteriorating the sensor response. This humidity effect is ascribed to the n-type doping effect or the adsorption of water molecules on the sensor surface, which alters the surface charge dynamics and interferes with gas adsorption processes.^67^ Water molecules undergo dissociation on the sensor surface, leading to the generation of hydroxyl species accompanied by the donation of electrons. The electron donation reduces the hole concentration in the p-type CNT/MoO_3_ nanohybrid, which decreases its electrical conductivity.^68,69^

The transient response curves and corresponding response levels of the CM15 sensor toward various ethanol concentrations (0.1–1 ppm) under different RH levels (0%, 30%, 45%, 60%, 75%, and 90%) at RT are presented in Fig. 11b and c. Water adsorption on the sensor surface alters the adsorbed species and lowers the response level. Specifically, the response of the CM15 sensor to 0.1 ppm ethanol gradually decreased from 22% to 14.6% as RH increased from 0% to 90%. These observations highlight that the sensor exhibits dominant ethanol-sensing characteristics at lower humidity levels. However, the dependence on humidity remains relatively weak for the nanohybrid sensor operating at RT, which is enabled by CNTs. CNTs demonstrate immunity to water molecule adsorption, contrasting with the pronounced humidity sensitivity observed in metal oxides as reported in the literature.^8,30,70–73^ This relatively low dependence on humidity represents an additional advantage of the CNT/MoO_3_ nanohybrid sensor for ethanol detection at ambient conditions.

## 4. Conclusion

The CNT/MoO_3_ nanohybrid was successfully synthesized using an arc-discharge method. The CNT content in CNT/MoO_3_ nanohybrids was tuned by adjusting the deposition time. Characterization through SEM, TEM, XRD, and BET analyses confirmed that the composites reveal a mesoporous structure, consisting of 0D MoO_3_ particles encapsulated on 1D CNTs. The CM15 sensor demonstrated the highest sensing performance in terms of response time, recovery time, and selectivity. The charge carrier transfer mechanism, involving both electrons and holes, is modified by the formation of a depletion layer in the nanohybrid. Compared to bare CNT and bare MoO_3_, the CNT/MoO_3_ nanohybrid exhibited a higher specific surface area, providing more surface-active sites, which enhanced its sensitivity. We believe that our nanohybrid materials show significant potential for the development of advanced ethanol detection sensors.

## Conflicts of interest

There are no conflicts to declare.

## Supplementary Material

RA-016-D6RA00372A-s001

## Data Availability

The data supporting this article have been included as part of the supplementary information (SI). Supplementary information is available. See DOI: https://doi.org/10.1039/d6ra00372a.
