# Supplementary material for: Arc-discharge-assembled CNT/MoO3 nanohybrids for ultra-sensitive and selective sub-ppm ethanol detection at room temperature
Source: RSC Adv. 2026 Mar 19;16(17):15464–76. doi: 10.1039/d6ra00372a (PMC13000908; doi:10.1039/d6ra00372a)
Supplement: RA-016-D6RA00372A-s001 [file RA-016-D6RA00372A-s001.pdf]

Supplementary Information (SI) for RSC Advances.
This journal is © The Royal Society of Chemistry 2026

Supporting Information

Arc-discharge-assembled CNT/MoO_3 nanohybrids for ultra-sensitive and selective sub-ppm ethanol detection at room temperature

Nguyen Minh Hieu[1], Cao Van Phuoc[2], Cao Viet Anh[3], Nguyen Manh Hung[5], Nguyen Duc Chinh[2], Vu Hai Trieu[1], Sutripto Majumder[2,6], Hoang Gia Chuc[1], Do Van Minh[2], Do Quang Trung[4], Tu Nguyen[4], Nguyen Van Du[4], Manh Trung Tran[1], Pham Thanh Huy[1], Jong- Ryul Jeong[2], Chunjoong Kim[2], Dojin Kim[2]*

[1]Faculty of Materials Science and Engineering, PHENIKAA School of Engineering, PHENIKAA University, Nguyen Trac, Duong Noi, Hanoi 12116, Vietnam

[2]Department of Materials Science and Engineering, Chungnam National University, Daejeon, 34134, Republic of Korea

[3]Department of Mechanical Engineering, Chungnam National University, Daejeon 34134, Republic of Korea

[4]Faculty of Fundamental Sciences, PHENIKAA School of Engineering, PHENIKAA University, Nguyen Trac, Duong Noi, Hanoi 12116, Vietnam

[5]Department of Materials Science and Engineering, Le Quy Don Technical University, Hanoi 100000, Vietnam

[6]Saveetha School of Engineering, Saveetha Institute of Medical and Technical Sciences (SIMATS), Chennai- 602105, Tamil Nadu, India

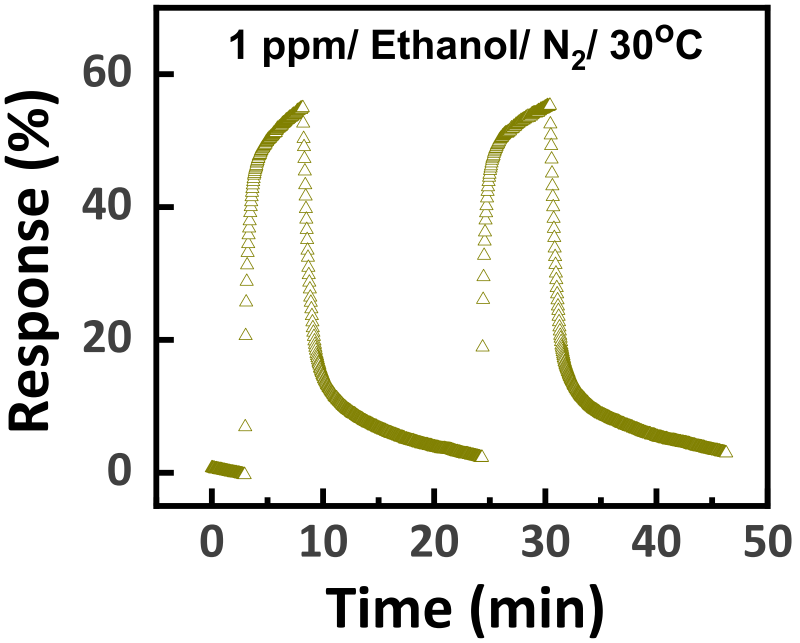

Fig. S1. The response behavior of CM15 toward 1 ppm ethanol exposure with N_2 as carrier gas.
